# Choroidal neovascularization secondary to half-dose photodynamic therapy for chronic central serous chorioretinopathy: A case report

**DOI:** 10.1097/MD.0000000000024790

**Published:** 2021-02-19

**Authors:** Zhengwei Zhang, Xiaona Bao, Zhifeng Wu, Jie Zhang

**Affiliations:** Department of Ophthalmology, The Affiliated Wuxi No. 2 People's Hospital of Nanjing Medical University, Wuxi, Jiangsu Province, P.R. China.

**Keywords:** central serous chorioretinopathy, choroidal neovascularization, photodynamic therapy, retinal pigment epithelium detachment

## Abstract

**Rationale:**

Half-dose or reduced-fluence photodynamic therapy (PDT) with verteporfin has been well acknowledged to be the most effective and permanent treatment with very low rates of complications. However, we report a case of chronic central serous chorioretinopathy (CSC) who developed choroidal neovascularization (CNV) secondary to half-dose PDT within only 3 weeks. Such an occurrence following this short a course of treatment has not been reported previously.

**Patient concerns:**

A 46-year-old Chinese man who had been diagnosed as acute more than 1 year ago revisited our department recently and complained of blurred vision again in his left eye.

**Diagnoses:**

Fluorescein fundus angiography (FFA) and indocyanine green angiography (ICGA) revealed patchy hyperfluorescent dots and optical coherence tomography (OCT) indicated irregular flat pigment epithelium detachment (PED) in the central macula. The patient was diagnosed with chronic CSC.

**Interventions:**

The patient was treated by half-dose PDT with verteporfin. Three weeks later, the patient complained of sudden blurred vision and fundus examination showed macular hemorrhages with a best-corrected visual acuity (BCVA) of 20/250. OCT angiography (OCTA) showed a distinct area of flower-like CNV located within the deep retinal slab. Secondary CNV had developed after a quite short course of half-dose PDT treatment. Subsequently, the patient was administered by 2 intravitreal injections of aflibercept (2 mg).

**Outcomes:**

Two months after the second intravitreal injection, macular hemorrhages and secondary CNV were completely resolved, and the BCVA improved to 20/25.

**Lessons:**

Patients of chronic CSC with irregular PED who undergo PDT should be warned of secondary CNV within a short course after treatment. If happened, it should be treated by intravitreal injections of anti-vascular endothelial growth factor agents as soon as possible.

## 1 Introduction

Central serous chorioretinopathy (CSC) is a common vision-threatening chorioretinal disease that causes idiopathic serous detachment of the retina, which primarily affects males aged 20 to 60 years.^[1]^ Pathogenesis of CSC is incompletely understood due to its multifactorial etiology and wide systemic associations. However, choroidal hyper-perfusion and hyperpermeability are known to play a major role.^[2]^ Photodynamic therapy (PDT) induces choroidal vascular remodeling and decreases choroidal permeability, and is advocated for the treatment of CSC.^[3]^

A widely reported complication of standard PDT is secondary choroidal neovascularization (CNV).^[4]^ Its mechanism is attributed to the pro-inflammatory effect, choriocapillaris occlusion, and significant reduction in chorioretinal perfusion caused by PDT. Subsequently, half-dose or reduced-fluence PDT with verteporfin has been well acknowledged to be the most effective and permanent treatment with very low rates of complications.^[5,6]^ Despite this, some rare but severe complications are inevitable.

Recently, a study reported high rates of CNV, detected using optical coherence tomography angiography (OCTA), associated with chronic CSC after half-dose PDT with a mean period of 39.5 months. ^[7]^ However, development of CNV, secondary to half-dose PDT for chronic CSC after a short course of treatment, is rare. Here, we report a patient of chronic CSC with serous pigment epithelium detachment (PED) whose visual acuity decreased abruptly due to CNV and macular hemorrhage, following a half-dose PDT, within 3 weeks of the intervention. Fortunately, this case was treated successfully with 2 intravitreal injections of aflibercept (2 mg).

## 2 Case presentation

More than 1 year ago, a 46-year-old Chinese man presented to our department with blurred vision in his left eye. At the time, he was diagnosed with acute central serous chorioretinopathy. Oral medications were prescribed; however, he was lost to follow-up. He had excellent uncorrected distance acuity (20/20 in both eyes). Records of optical coherence tomography (OCT) B-scan showed neurosensory detachment at the central macula.

He revisited our department recently and complained of blurred vision again since 1 month. At this visit, he underwent a complete ophthalmic examination, including slitlamp biomicroscopy, best-corrected visual acuity (BCVA), non-contact tonometry, detailed fundus examination, fluorescein fundus angiography (FFA), indocyanine green angiography (ICGA), OCT, and OCTA.

**Figure 1:**
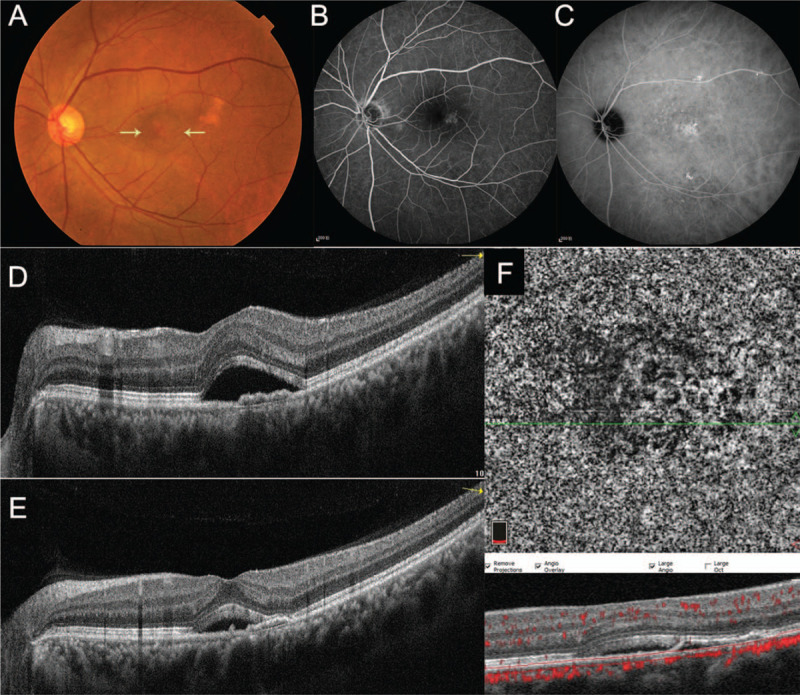
Multimodal imaging before treatment Fundus photograph showing a shallow sensory detachment in the macula (yellow arrows) in the left eye (A). The lesion revealed patchy hyperfluorescent dots temporal to the fovea in the late phase of FFA (B) and ICGA (C). OCT B-scan showed a dome-shaped serous detachment of the neurosensory retina and a flat serous PED at both the initial visit (D) and this visit (E). OCTA did not detect a distinct CNV (F).

The BCVA was 20/20 OD and 20/25 OS. Intraocular pressure was within normal limits, and anterior segment examination was unremarkable in both eyes. Fundus examination of the right eye was normal, but the left eye showed a shallow sensory detachment in the macula (Fig. 1A). On FFA, the lesion revealed patchy hyperfluorescent dots temporal to the fovea in the early and late phases (Fig. 1B). ICGA also revealed patchy hyperfluorescent dots in the middle and late phases (Fig. 1C). OCT B-scan showed a dome-shaped serous detachment of the neurosensory retina and a flat serous PED at the first visit (Fig. 1D) and subsequent visit (Fig. 1E). OCTA, reported to be useful for identifying hidden CNVs that could not be found via FA and ICGA, did not show a distinct CNV (Fig. 1F).

**Figure 2:**
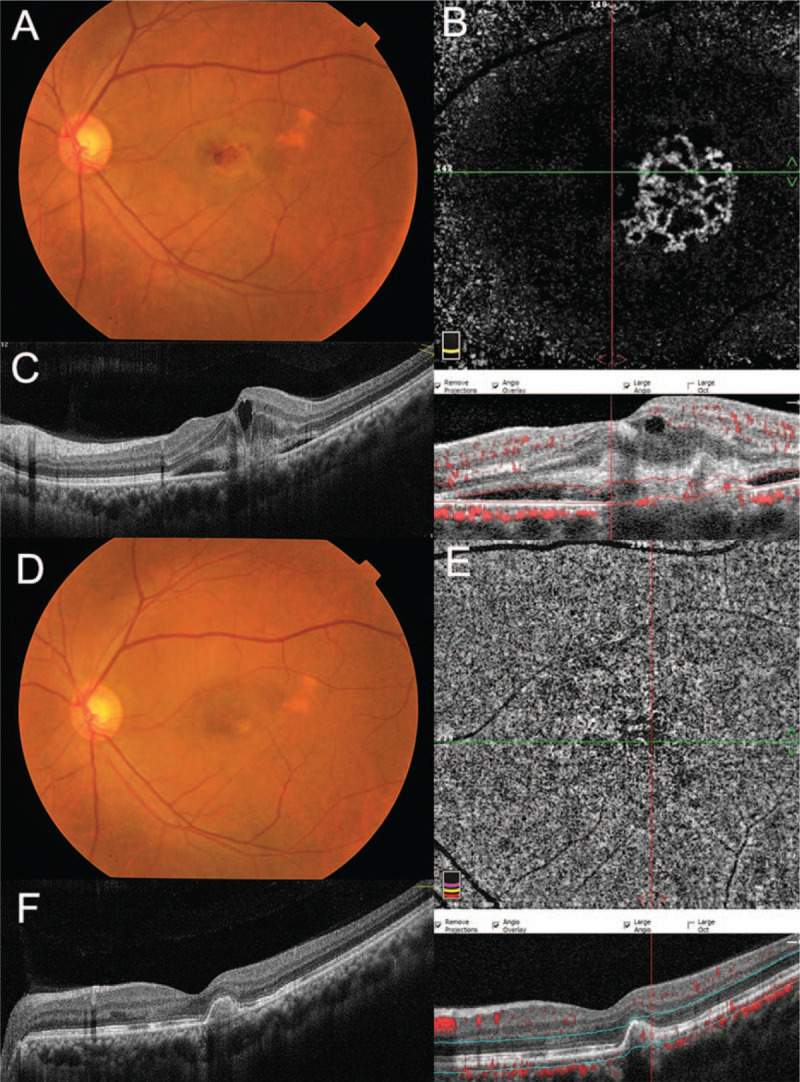
Multimodal imaging after half-dose PDT Fundus photograph showing macular hemorrhages approximately 3 weeks after treatment (A). OCTA showing a distinct area of flower-like CNV located within the deep retinal slab (B). OCT B-scan displaying subretinal hyperreflective material corresponding to the CNV complex located above the RPE (C). Three months after two intravitreal injections of aflibercept (2 mg), macular hemorrhages were completely resolved in the fundus (D) and the CNV did not recur in the OCTA image (E), but a small PED was still present on the OCT B-scan (F).

The patient was diagnosed with chronic CSC. Treatment employed was half-dose PDT with verteporfin. Fifteen minutes after the start of the intravenous infusion of verteporfin (3 mg/m^2^), a 689 nm laser was delivered (600 mW/cm^2^; 83 s). The delivered radiation covered the hyperfluorescent area of the corresponding serous subfoveal PED in the middle or late phase of the ICGA. Three weeks later, the patient complained of sudden blurred vision and revisited our department. Fundus examination showed macular hemorrhages (Fig. 2A) with a BCVA of 20/250. OCTA showed a distinct area of flower-like CNV located within the deep retinal slab (Fig. 2B). OCT B-scan revealed subretinal hyperreflective material corresponding to the CNV complex located above the retinal pigment epithelium (RPE) (Fig. 2C). Unfortunately, secondary CNV had developed after half-dose PDT in this case.

Next day, intravitreal injection of aflibercept (2 mg) was administered. One month later, the patient's symptoms were relieved and his BCVA improved to 20/100. Fundus examination revealed almost complete resolution of macular hemorrhages. OCTA showed the CNV had completely disappeared. To consolidate treatment, a second intravitreal injection of aflibercept (2 mg) was administered. Two months later, the BCVA improved to 20/25. Macular hemorrhages were completely resolved in the fundus (Fig. 2D) The CNV did not recur in the OCTA image (Fig. 2E), but a small PED was present on the OCT B-scan (Fig. 2F).

## 3 Discussion

Although PDT with verteporfin was originally developed for treating CNV secondary to age-related macular degeneration, it was soon used as an important treatment modality for chronic CSC.^[6,8]^ Full-dose PDT with verteporfin (6 mg/m^2^ intravenously) used in CSC may sometimes, cause severe complications that are unacceptable for a disease with relatively favorable prognosis. Verteporfin dye might accumulate selectively around the choroidal hyper-permeable area owing to slow blood flow and vascular congestion, which may lead to irreversible occlusion of the choroidal vessels.^[9]^ Additionally, PDT may cause RPE alterations and induce the release of vascular endothelial growth factor (VEGF), contributing to the development of CNV.^[10]^

Many studies have recommended half-dose or reduced-fluence PDT for treating CSC. ^[5]^ More recently, Wu et al^[7]^ reported high rates of CNV associated with chronic CSC after half-dose PDT with a mean period of 39.5 months (range: 4–138 months) from treatment to OCTA examination. However, the development of CNV secondary to half-dose PDT within 1 month of treatment is rare. Hwang et al^[11]^ reported 1 such case of chronic CSC developed secondary CNV and subretinal hemorrhages 1 month after reduced-fluence PDT (30 mJ/cm^2^). In their case, the PED was small and dome-shaped under the fovea before treatment. Fortunately, their patient recovered his vision after 2 intravitreal bevacizumab injections.

In our case, we employed half-dose PDT (3 mg/m^2^), reported to result in favorable outcomes with less risk of complications.^[5]^ Unfortunately, secondary CNV developed 3 weeks post-treatment. To the best of our knowledge, such an occurrence following this short a course of treatment has not been reported previously. Although the pathophysiology of CSC is poorly understood, it is reasonable to apply optimized PDT at the area of choroidal congestion and RPE leakage, thus preventing the disease from developing. Choriocapillary thinning secondary to the underlying choroidal congestion, long-standing serous PED, and pre-existing defects in Bruch's membrane due to chronic RPE changes, may be a risk factor for the development of CNV. Demircan et al^[12]^ reported that choriocapillaris perfusion seemed to decrease in the very early period following half-fluence PDT and returned to normal after 1 month of therapy. Therefore, half-dose PDT may further exacerbate the already compromised choriocapillaries and increase the incidence of CNV. Undoubtedly, the risk does not outweigh the benefits of optimized PDT for the treatment of CSC. However, patients undergoing PDT with relatively good vision should be warned of this rare complication.

Another rare complication secondary to PDT, which needs to be distinguished from secondary CNV, is PDT-induced acute exudative maculopathy (PAEM).^[13]^ PAEM is defined as a massive subretinal serofibrinous exudation with or without acute severe vision impairment.^[14]^ It occurs within days after PDT but has a self-resolving course and favorable prognosis. PAEM is rarely reported after treatment of chronic CSC, with only 3 cases reported in the literature. The pathogenesis includes breakdown of the blood-retinal barrier, RPE pump dysfunction, and inflammatory surge of VEGF occurring after PDT. OCTA is a useful tool to distinguish PAEM from CNV.

To summarize, CNV secondary to half-dose PDT for chronic CSC after a quite short course of treatment is rare. Considering an otherwise favorable prognosis for CSC treatment, patients with chronic CSC who undergo PDT should be warned of this rare complication. Fortunately, it can be successfully treated by intravitreal injection of anti-VEGF agents.

## 4 Disclosure

The Institutional Review Board of the Affiliated Wuxi No.2 People's Hospital of Nanjing Medical University approved the protocol, and our study was performed in accordance with the tenets of the Declaration of Helsinki. Written informed consent was obtained from the patient for publication of this case report and all accompanying images. There is no conflict of interest exists in the submission of this manuscript, and manuscript is approved by all authors for publication.

## Author contributions

ZWZ, XNB and JZ collected the clinical information of the patient, analyzed and interpreted the clinical data. ZWZ wrote the drafting of this manuscript. ZFW and JZ reviewed and edited the manuscript. JZ was responsible for supervision. All authors read and approved the final manuscript.

**Conceptualization:** Zhengwei Zhang, Xiaona Bao, Zhifeng Wu, Jie Zhang.

**Funding acquisition:** Zhengwei Zhang.

**Supervision:** Jie Zhang.

**Validation:** Zhengwei Zhang, Xiaona Bao, Zhifeng Wu, Jie Zhang.

**Writing – original draft:** Zhengwei Zhang.

**Writing – review & editing:** Zhifeng Wu, Jie Zhang.
